# Recent Advances in the Therapeutic Effects and Molecular Mechanisms of Baicalin

**DOI:** 10.3390/biology14060637

**Published:** 2025-05-30

**Authors:** Xiaoyuan Qiu, Renyin Huang, Junke Xie, Shanshan Luo, Xiang Cheng, Jing Cui, Desheng Hu

**Affiliations:** 1 Department of Integrated Traditional Chinese and Western Medicine, Union Hospital, Tongji Medical College, Huazhong University of Science and Technology, Wuhan 430022, China; qxyuan8@foxmail.com; 2 Jingshan Union Hospital, Union Hospital, Huazhong University of Science and Technology, Wuhan 430022, China; jmjshry@sina.com (R.H.); xjkwudi@sohu.com (J.X.); 3 Institute of Hematology, Union Hospital, Tongji Medical College, Huazhong University of Science and Technology, Wuhan 430022, China; shsh689@126.com; 4 Department of Cardiology, Union Hospital, Tongji Medical College, Huazhong University of Science and Technology, Wuhan 430022, China; nathancx@hust.edu.cn; 5 Health Management Center, Hubei Provincial Hospital of Integrated Chinese and Western Medicine, Wuhan 430015, China

**Keywords:** traditional herbal medicine, flavonoids, baicalin, anti-inflammation, antioxidation

## Abstract

Baicalin, a kind of polyphenolic flavonoid, is a major bioactive flavone derived from the root of *Scutellaria baicalensis*, which has been widely utilized in clinical practice in China for thousands of years. In recent years, it has attracted increasing attention due to its potential therapeutic properties observed in preclinical studies involving various disease models. However, the precise mechanisms underlying its biological activities have not been fully elucidated. This review summarizes recent research progress on the molecular mechanisms through which baicalin exerts its effects, particularly in tumor suppression, cardiovascular protection, neuronal preservation, and glucose and lipid metabolism regulation in murine models. Additionally, we discuss the delivery methods of baicalin and its transformation by intestinal microbiota.

## Simple Summary

Natural products are one of the most important sources of drug discovery, providing abundant structural templates and inspiration for new drug development. They play a vital role in modern pharmaceutical research. Traditional Chinese medicine (TCM), known for its unique therapeutic effects, has been used in China for centuries. Increasing attention has been paid to the study and development of its main active constituents. This review focuses on baicalin, a key bioactive compound derived from TCM, and systematically summarizes recent advances in its pharmacological effects and underlying mechanisms in the treatment of cancer, cardiovascular diseases, neuroprotection, and metabolic disorders, aiming to provide valuable insights and references for future research.

## 1. Introduction

Traditional Chinese medicines (TCMs) are invaluable resources due to their proven clinical effects and complex components, which confer a wide range of therapeutic functions. However, this complexity also introduces uncertainty about the specific actions of these herbs, and the imprecise target of herbal molecules complicates the explanation of their clinical outcomes. Advances in modern medical science now allow us to isolate the main bioactive components and elucidate the underlying mechanisms. More importantly, TCMs have the potential to provide broad ground for modern drug development [1].

Huangqin (*Scutellaria baicalensis*), with its bitter taste and mild nature, is one of the most common traditional Chinese herbs. It has been historically employed for removing damp heat, quenching fire, counteracting toxicity, arresting bleeding, and preventing abortion according to traditional Chinese medicine theory. It has also been widely used in Chinese herbal formulas together with other herbs (Table 1).

**Figure 1:**
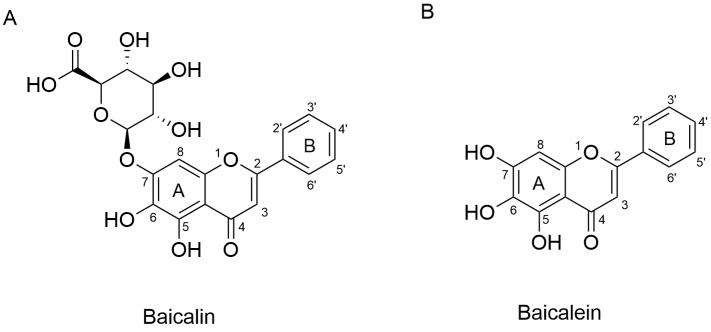
Chemical structures of baicalin (**A**) and baicalein (**B**).

Advances in modern pharmacology have raised expectations for the precise identification of active constituents in TCMs. To date, more than 200 chemical constituents have been isolated and identified from *Scutellaria baicalensis*, the majority of which are flavonoids and their glycosides [20]. Utilizing chromatographic fingerprint analysis and a backpropagation–artificial neural network model, baicalein, baicalin (Figure 1), wogonoside, and wogonin were determined as the quality control markers of *Scutellariae radix* and its wine-processed and carbonized products [21]. While all four flavonoids have been studied, this review focuses specifically on baicalin, which is the most abundant constituent, accounting for approximately 25.80% of the methanolic extract of *Scutellariae radix* and 8.12% of the dry root mass [22]. Given the complexity of herbal extracts, it is often difficult to ascribe specific biological effects to baicalin alone. Thus, in order to provide a more accurate evaluation of baicalin’s pharmacological properties, this review focuses on studies in which baicalin has been isolated and its concentration clearly determined.

Baicalin is a free-B-ring flavonoid purified from *Scutellariae radix* through uridine diphosphate glucuronidation. Purified baicalin (baicalein 7-*O*-glucuronide) is a light yellow-to-yellow powder with a molecular weight of 446.3640. It is barely dissolvable in aqueous buffers, so its oral bioavailability is low. As a study reported, the absolute bioavailability of oral baicalin is 30% [23], and numerous studies have been conducted with the aim of improving its intestinal absorption [24,25].

**Figure 2:**
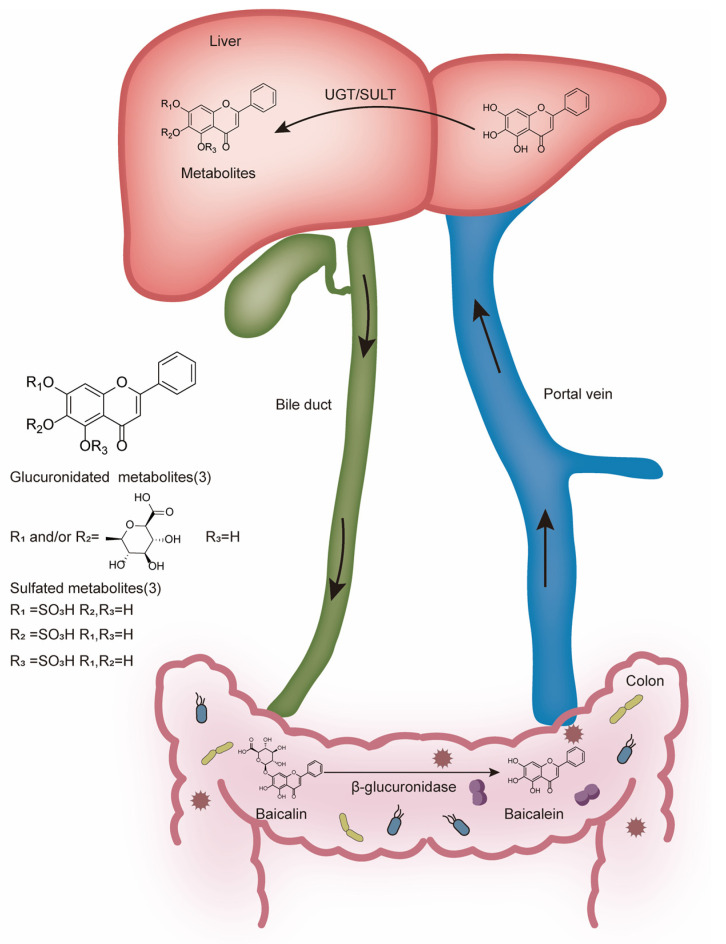
Metabolism of baicalin in the colon and liver.

Baicalin is an important class of flavonoid glycosides, and baicalein is the aglycone of baicalin. Baicalin is metabolized to baicalein via β-glucuronidase produced from intestinal microbiota, indicating that the digestive microbiota plays an important role in the biological activity of baicalin [26]. In fact, bacterial hydrolysis might be the only way and the rate-limiting step for its absorption [27]. Glucuronidation is the main metabolic pathway for baicalin. The absorbed baicalein is widely metabolized in the liver by two phase II metabolic enzymes, UDP glucuronosyltransferase (UGT) and sulfotransferase (SULT). UGT catalyzes the transfer of glucuronic acid to lipophilic substrates [28], and SULT catalyzes the transfer of the sulfate groups. Then, the glucuronides (including baicalin) [29] or sulfates [30] of baicalein enter the enterohepatic circulation (Figure 2). Following this process, baicalin is distributed to different tissues such as the brain, lung, heart, and so on. Oral administration of baicalin exclusively presents in the plasma as baicalein glucuronides/sulfates, which means that the conjugated metabolites of baicalein are in fact responsible for its in vivo effects [31]. In vitro studies on baicalin only partially explain its in vivo effects. Eventually, baicalin is primarily excreted into the bile in the form of glucuronides. Of note, the enterohepatic circulation contributes to the overall systemic disposition of baicalin and its conjugated metabolites [23].

Baicalin has been reported to possess multifaceted functions, including anti-inflammatory, anti-bacterial, antiviral, and antiallergic effects. Over the past decade, the antioxidant and anti-inflammatory activity of baicalin has been given considerable attention. Considering the increased attention paid to herbal medicines and the promising effects of baicalin, we discuss the diseases in which baicalin may have beneficial effects. We also addressed the pathways by which baicalin participates in exhibiting its effects (Table 2).

## 2. Antitumor Effects

In recent years, there has been an increase in the development of natural anti-cancer compounds due to their therapeutic efficacy, fewer side effects, and lower cost [71]. Early studies focused on formulations containing baicalin. Dating back to the 1990s, Japanese scientists conducted a considerable amount of research on sho-saiko-to, which was the most popular herbal medicine and contained huangqin as the main component, and confirmed its effect on suppressing hepatocellular carcinoma and cholangiocarcinoma in vitro [72,73]. Another study pointed out that sho-saiko-to displayed antitumor and anti-metastatic effects on melanoma in vivo and in vitro [74]. However, formulations make it difficult to attribute the effects directly to baicalin, and studies on purified baicalin have been increasingly focused. The direct anticancer activities of baicalin have been confirmed in prostate cancer, lung cancer, lymphoma, and hepatocellular carcinoma (HCC) [32,33,75,76,77]. And multiple signaling pathways are involved, including apoptosis, the cell cycle, invasion, migration, angiogenesis, autophagy, and immune evasion [78,79]. Sometimes it seems to be controversial across different studies. For example, research conducted by Su’s group tested concentration-dependent cell growth inhibition in response to baicalin (50–200 μmol/L) in breast cancer cells MCF-7 and MDA-MB-231, and the effect was enhanced when in combination with baicalein [80]. While another study found that baicalin (100 μg/mL, 224 μmol/L) enhanced the growth of MCF-7 human breast cancer cells, but baicalein and wogonin significantly inhibited MCF-7 cell growth [81]. These inconsistent findings could be due to the differences in cell line sensitivities, cell states, experimental conditions, or the concentration of baicalin. Such discrepancies emphasize the need to test different drug concentration gradients and multiple cell lines. In most studies, this direct antitumor effect is mainly accomplished through apoptosis, cell cycle arrest, and ferroptosis [41]. Dou et al. illustrated that baicalin treatment dramatically inhibited tumor growth not by inducing apoptosis but through the induction of tumor cellular senescence; moreover, they established decidual protein induced by progesterone as a key node regulating senescence induction, which could be a novel target for cancer treatment [34]. And in a recent study, researchers found that autophagy is also a strategy by which baicalin inhibits tumor growth [82]. Except for the inhibition of cell growth and proliferation, baicalin exerts its antitumor effects by inhibiting migration and invasion [83,84], which plays a vital role in the progression of cancer and is usually associated with poor prognosis. Furthermore, this study also demonstrated that baicalin suppressed HCC cell growth and metastasis by inhibiting ROCK1 signaling, which regulates cell polarity and migration by boosting actomyosin contraction and focal adhesions, and ROCK1 might be a stable and direct target of baicalin [40]. This indicates that baicalin not only acts through broad cytotoxic mechanisms but also targets specific regulatory molecules of metastasis, suggesting good therapeutic selectivity.

Tumor progression is also modulated by the tumor microenvironment (TME), which is the home of cancer cells and serves as a bridge connecting cancer with the entire organism [85]. The TME is populated by many immune cells, of which macrophages are among the most abundant [86]. Generally, macrophages are classified into M1 and M2. M1 is often considered antitumoral, and M2 is typically regarded as pro-tumoral. There are several studies demonstrating that baicalin mediates tumor-associated macrophage (TAM) repolarization and suppression of tumor progression. Feng’s group measured M1/M2-like macrophages in the liver of baicalin-treated mice, and they found that baicalin treatment led to an increase in the M1-like macrophage population while there was a significant reduction in the M2-like population in the liver tissue of the mice. This was further evidenced by the fact that baicalin skewed M2-like macrophages toward the M1-like phenotype, without causing any significant changes in M1-like macrophages in vitro [33]. Also, in non-small cell lung cancer, baicalin effectively inhibited tumor growth in Lewis lung carcinoma tumor-bearing mice and increased the infiltration of M1-type macrophages in the TME [87]. When combined with a nano-complex, the baicalin taken up by TAMs was released to the tumor sites, and it not only killed tumor cells directly but also remodeled the tumor microenvironment, as mentioned above [88]. Except for macrophages, baicalin also induces responses in T and B cells [89]. Nonetheless, studies on its immunomodulatory functions beyond macrophages are inadequate, and more research is needed to clarify whether baicalin can support long-term antitumor immunity.

Despite the considerable advances that have been made in cancer treatment, chemotherapy remains widely used. However, drug resistance is an obstacle to clinical effectiveness. Studies have shown how baicalin works in chemotherapeutic resistance. In breast cancer cell lines, baicalin was able to enhance doxorubicin cytotoxicity via the reactive oxygen species (ROS)/[Ca^2+^]-mediated intrinsic apoptosis pathway [90]. Consistent with this, baicalin successfully enhanced sensitivity to 5-Fluorouracil and reduced Ehrlich tumor growth (a spontaneous murine mammary adenocarcinoma) via cooperative inhibition of inflammation, angiogenesis, and triggering apoptotic cell death [38]. Also, an efficient in vitro and in vivo study in colorectal cancer showed that baicalin enhanced the effect of 5-fluorouracil-based chemotherapy via inhibition of the CDK-RB pathway [39]. Studies in gastric cancer cell lines have provided evidence that baicalin enhances 5-Fluorouracil by promoting ROS-related ferroptosis in gastric cancer and inhibits drug resistance [42]. These findings support the potential of baicalin as a chemosensitizer, but the safety and pharmacokinetics of baicalin in combination therapy need to be evaluated systematically.

Taken together, baicalin effectively inhibits the growth of various cancer cells and leads to tumor shrinkage. On the one hand, it kills cancer cells and inhibits proliferation; on the other hand, it plays a role in immune cells and remodels the tumor microenvironment. More importantly, when in combination with other chemotherapeutic drugs, 5-fluorouracil, for instance, it alleviates chemotherapy resistance while retaining its own antitumor function at the same time.

## 3. Cardiovascular Protection Effects

Cardiovascular diseases (CVDs) are the leading cause of death globally. An estimated 17.9 million people died from CVDs in 2019, representing 32% of all global deaths. Baicalin has been reported to improve hyperglycemia-induced dysplasia of the cardiovascular system during early embryo development. In early chick embryos, it rescued hyperglycemia-induced cell proliferative reduction and apoptosis increase, redressed the unbalanced secondary effect of autophagy on heart tube formation, and stabilized the oxidative stresses’ secondary effect on angiogenesis, thus reversing the hyperglycemia-inhibited development of early chick embryos [91]. Herein, we review the protective effects and underlying pharmacological mechanisms of baicalin against CVDs. The available studies suggest that baicalin has great potential for the treatment of CVDs and is worthy of more research.

### 3.1. Protection of the Heart

Myocardial ischemia is a main factor that leads to the loss of cardiomyocytes. In hypoxia/reoxygenation (H/R)-treated neonatal rat cardiomyocytes, baicalin pretreatment reduced cell death, attenuated oxidative stress, and improved morphological changes; what is more, it suppressed inflammatory cytokine IL-6, increased anti-inflammatory cytokine IL-10 levels, and inhibited the nuclear translocation of NF-κB induced by H/R. These results indicate that baicalin has a positive effect on cardiomyocytes suffering from H/R insults through antioxidation and anti-inflammation mechanisms [92]. Furthermore, the protective effect was verified in vivo. Baicalin improved cardiac function, decreased the area of myocardial infarction, and inhibited the apoptosis of myocardial cells in myocardial ischemia–reperfusion (IR) rats, and the protective mechanism was related to the promotion of NO production and the inhibition of necroptosis in cardiac microvascular endothelial cells through activation of the PI3K-AKT signaling pathway [93]. Zeng et al. conducted a meta-analysis of animal studies on the positive pharmacological effects of baicalin on IR injury, illustrating a chain of preclinical evidence and providing rigorous and systematic support for further clinical research [94].

Another cardiomyocyte disorder is myocardial hypertrophy, which is a compensatory response to a persistent increase in load. In both angiotensin II (Ang II)-induced cardiomyocyte hypertrophy in vitro models and abdominal aortic constriction-induced mouse cardiac hypertrophy in vivo models, baicalin exerted an anti-cardiac hypertrophy effect. Molecular docking experiments indicate that baicalin might directly interact with PSMB5, inhibiting the activation of the proteasome and the degradation of SIRT3, whose downstream signaling pathway has an impact on cardiac hypertrophy [45].

### 3.2. Regulation of Blood Vessels

The effect of baicalin in regulating blood vessels is reflected in multiple aspects, including anti-atherosclerosis, angiogenesis, and vasodilation. Atherosclerosis is a chronic inflammatory disease of the arterial wall, with an imbalanced lipid metabolism and a maladaptive immune response involved [95]. The excessive proliferation and migration of vascular smooth muscle cells (VSMCs) are important events in the development of atherosclerotic lesions. Baicalin induced growth arrest in platelet-derived growth factor (PDGF)-BB stimulated VSMCs via blockade of the PDGF receptor β-ERK1/2 signaling cascade. In rat carotid arterial balloon-injury models, it also prevented neointimal hyperplasia [96]. Also, Jin’s group reported that baicalin exerted anti-atherosclerosis effects through regulation of the lipid profile. In THP-1 cells, decreased ox-LDL-induced foam cell formation and intracellular lipid accumulation were observed at nontoxic concentrations [43]. Similarly, an in vivo study demonstrated that baicalin decreased lipid accumulation by upregulating the lipolysis-related proteins peroxisome proliferator-activated receptor α(PPARα) and carnitine palmitoyl-transferase1 (CPT1) and suppressing adipogenesis-related proteins sterol-CoA response element binding protein-1c (SREBP-1c) and ACS; in addition, lower expression of LDL-C and TG and higher concentrations of TCH were detected in the baicalin model group compared with the AS group. Except for the anti-adipogenic effect, antioxidant and anti-inflammatory effects also participate in anti-atherosclerotic action [97]. In one study, it was shown that baicalin exhibited potent biological activity to restore the function of endothelial cells and inhibited VSMC proliferation and migration and the release of inflammation markers from activated macrophages [98]. These studies collectively suggest that baicalin exerts multi-target anti-atherogenic potential, likely through a combination of endothelial restoration, lipid regulation, and inflammation suppression.

Angiogenesis plays a critical role in injury caused by ischemia, like stroke and myocardial infarction. Vascular endothelial growth factor (VEGF) is one of the most specific factors that stimulate angiogenesis. Through the activation of the ERRα, baicalin induced VEGF expression and angiogenesis [44]. This underscores baicalin’s therapeutic promise in ischemic cardiovascular conditions by promoting vascular regeneration.

Hypertension, characterized by elevated systemic arterial pressure, represents a major risk factor for cardiovascular diseases and remains the leading cause of premature death worldwide [99]. Vascular constriction/relaxation function directly affects blood pressure. Influx of extracellular Ca^2+^ regulates the contraction of smooth muscle; the major pathway for this increase is through voltage-dependent Ca^2+^ channels (VDCCs). Large-conductance Ca^2+^-activated channels, acting as a negative feedback mechanism, play a central role in the regulation of vascular tone [100]. Baicalin showed VDCC inhibition and BK activation properties by stimulating the cGMP/PKG and cAMP/PKA pathways [101]. Also, baicalin effectively decreased vascular tension in spontaneously hypertensive rats’ (SHR) aortas and lowered their blood pressure. The activated ATP-sensitive potassium channel is, in part, explained by the vasorelaxant effect of baicalin [102]. RNA sequence analysis was conducted to explore the underlying mechanisms of baicalin on hypertension, and the calcium signaling pathway and vascular smooth muscle contraction signaling pathways were found. Through a series of experiments, Peng’s group not only verified that baicalin reversed the elevation of BP, vascular pathological injury, and VSMC proliferation induced by Ang II, but also demonstrated that the mechanism was due to the activation of the MLCK/p-MLC signaling pathway and decreased intracellular calcium release in VSMCs [47]. These findings collectively indicate that baicalin improves hypertension mainly through the synergistic regulation of vascular tone and calcium-dependent contractile machinery.

## 4. Neurological Disorders

Brain disorders such as cerebral ischemia and neurodegenerative diseases have become the biggest threats to people [103]. Emerging data indicate the potential neuroprotective function of baicalin in the treatment of these diseases. In vitro, baicalin showed protective effects on PC12 cells suffering from colistin sulfate-induced apoptosis [104]. For brain diseases, effective blood–brain barrier (BBB) penetration is a formidable challenge [105]. Evidence from multiple studies supports the potential of baicalin passing through the BBB [106], which is the basis for its effect on the central nervous system. Recently, many studies have focused on borneol–baicalin liposome [107,108]. Borneol is widely used for “waking up” the brain according to TCM theory. As expected, the addition of borneol prolonged the efficacy time of baicalin, improved blood–brain barrier integrity, and better exerted the therapeutic effect on cerebral I/R injury in vivo and in vitro. Improving the bioavailability of baicalin can also be achieved by transforming the method of drug administration. Nose-to-brain drug delivery administration is an alternative way for baicalin to be used to treat brain diseases [109]. Baicalin-loaded ligand-modified nanoparticles were prepared for nose-to-brain delivery, and the effect was significant because baicalin was delivered to the entire brain with little delivery to the peripheral circulation [110].

### 4.1. Neuroprotection in Ischemic Stroke

Stroke is a leading cause of mortality and disability worldwide. Ischemic stroke caused by arterial occlusion is responsible for the majority of strokes. Increasing knowledge about the exact pathophysiology of stroke is necessary to design suitable drugs. The ischemic cascade responses in cells include reduced availability of glucose and oxygen, increased extracellular levels of glutamate, neuronal calcium influx (mediated via the *N*-methyl-d-aspartate (NMDA) ion receptor (NMDAR)) and subsequent production of nitric oxide by neuronal nitric oxide synthase (NOS), blood–brain barrier dysfunction, and pro-inflammatory cytokine release from microglia, leading to an inflammatory response; the result of all of these cumulative effects is neuron death. Interruption of these processes may provide a way to prevent, or at least reduce, the ischemic damage. Management of ischemic stroke focuses on rapid restoration of blood flow with intravenous thrombolysis and endovascular thrombectomy, and this is critical to reduce disability [111]. Tissue plasminogen activator is the only FDA-approved drug for the treatment of cerebral ischemia. Together with this, neuroprotective therapy represents another major strategy for ischemic stroke [112]. Advances in the natural flavonoid baicalin are highlighted in improving neuroprotection following brain ischemia injury.

The authors of a previous study assessed the efficacy of baicalin in rat models of cerebral artery occlusion, and neurological deficits, cerebral infarct volume, and pathomorphological change in ischemic brain tissue were assessed. These three indicators were all improved in the baicalin group compared with the ischemia group; also, cerebral infarct volume was similar in the valproic acid (positive control) and baicalin groups [113]. These findings suggest that baicalin appears to be a potential therapeutic agent for the treatment of ischemic stroke. Considerable effort has been made to identify the targets of baicalin and the pathways it regulates. In nuclear magnetic resonance titration experiments, baicalin displayed high PDZ2 binding affinity, which is associated with NMDAR [114]. Gene expression profiling was conducted to identify the differential gene expression and the pharmacological mechanism of baicalin in cerebral ischemia rats. By comparing the differences in infarction areas before and after baicalin treatment, cell signal transduction and protein phosphorylation were significant [115]. After improving the research method, Wang et al. used transcriptome analysis to explore the pure therapeutic mechanisms of baicalin contributing to phenotype variation and the reversal of pathological processes in ischemic stroke mice. According to the analysis, seven specific targeted molecules were found; they are ATF3, BCL2L1, ARF1, FGF12, GRIN1, MAP2K6, and PRKAR1E. Forty-one differentially expressed genes were identified. Based on these genes, a statistically significant network was constructed, and the functions of the target network include cell death, genetic disorder, and immunological disease. Fifty-one canonical pathways and seventy biological functions were identified. This pure mechanistic analysis might provide a clearer outline of the target profiles of baicalin therapies [116].

Glutamate is considered the triggering spark in the cascade of responses of ischemic neuronal damage. Its level was increased with excessive neurological stimulation, causing glutamate-induced neuronal toxicity and excitotoxicity [117]. In the process of glutamate clearance, Na^+^-dependent high-affinity glutamate transporters (excitatory amino acid transporters, EAATs) remove glutamate from the extracellular space to maintain its extracellular concentration below excitotoxic levels. Of all five EAATs, EAAT2 (GLT-1), the glial-type glutamate transporter, provided the majority of total glutamate uptake. Moreover, glutamine synthetase (GS) in astrocytes was required for the glutamate–glutamine cycle and helped glutamate be repackaged into synaptic vesicles [118]. Baicalin might help with the clearance of glutamate via different mechanisms. It was confirmed to upregulate GLT-1 expression in peri-infarct cortices in hypoxic–ischemic encephalopathy models 24 h after injury and exhibited protective effects [119]. Also, baicalin was found to maintain GS protein stability from 20S-mediated proteasomal degradation in astrocytes [120]. These results provide evidence to support baicalin’s ability to combat glutamate excitotoxicity to prevent ischemic neuronal injury.

The inflammatory response to cerebral ischemia is another important process in stroke pathobiology and neuronal death. 5-lipoxygenase is a key enzyme in the catalytic conversion of arachidonic acid to inflammatory mediator leukotrienes. Studies in oxygen–glucose deprivation-induced neuronal damage indicate that baicalin inhibits 5-lipoxygenase activation mediated by NMDAR and oxidative stress [121,122]. Moreover, Chen’s results demonstrated the neuroprotective effect of baicalin on permanent middle cerebral artery occlusion (pMCAO) rat models, and this protection might be associated with its potent anti-inflammatory and antiapoptotic properties. In further detail, mRNA expression of iNOS and COX-2 in the ischemic brain after pMCAO decreased after baicalin treatment [123]. In another study, the effects of baicalin on the TLR2/4 signaling pathway were investigated. Both in the oxygen glucose deprivation model in vitro and the I/R model in vivo, TLR2/4 responded to the damage, and the expression of its downstream factor, tumor necrosis factor α (TNFα), increased. As for NF-κB, baicalin not only decreased its expression but also inhibited its translocation from the cytoplasm to the nucleus in vitro [124]. These studies show that the protective role of baicalin in ischemic neuronal injury is closely related to its anti-inflammatory properties.

**Figure 3:**
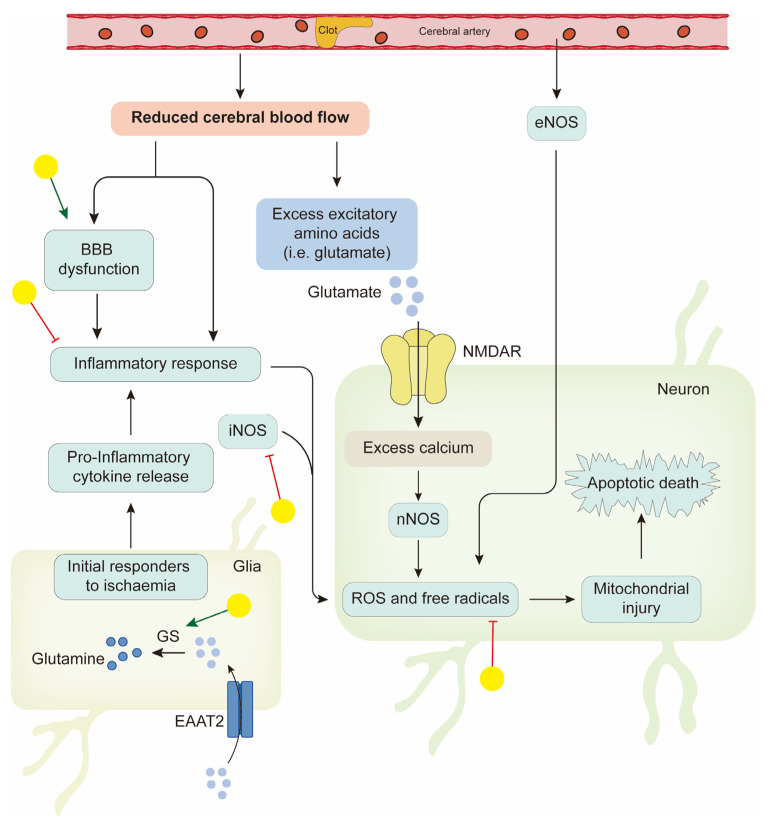
Neuroprotective effects of baicalin in ischemic stroke. Yellow balls indicate baicalin.

The disruption of the BBB is also a factor that aggravates the brain damage observed in cerebral IR injury. Baicalin has been found to be capable of restoring the barrier function of the BBB in various conditions [125,126]. Therefore, baicalin can preserve brain tissue viability before reperfusion through anti-excitotoxicity and anti-inflammatory effects, protection of the BBB’s integrity properties, etc. (Figure 3).

### 4.2. Neuroprotection in Neurodegenerative Diseases

A wide spectrum of neurodegenerative disorders, such as Alzheimer’s disease (AD) and Parkinson’s disease (PD), affect the central nervous system (CNS). Characterized by the progressive degradation of synapses and axons, they eventually lead to neuronal death [127]. Baicalin can act as an iron chelator to protect dopaminergic neurons and delay PD neural degeneration in Parkinson’s disease rats [48]. In C6 glioma cells, baicalin decreased divalent metal transporter1 expression, increased ferroportin1 expression, subsequently lowered the iron concentration, and protected nerve cells [128]. As for AD, the improvement of cognitive function and brain damage and decreased eicosanoid production were observed in mice models of flavocoxid (a mixture of purified baicalin and catechin), and these protective effects could be attributed to its anti-inflammatory and anti-apoptotic properties [49]. Analogous to the BBB, the blood–spinal cord barrier (BSCB) is a specialized protective barrier that plays a crucial role in maintaining the homeostasis and internal environmental stability of the CNS [129]. Disruption of the BSCB is common in neurodegenerative diseases and CNS traumatic injury. In an established spinal cord injury rat model, baicalin evidently restored BSCB integrity. And in SH-SY5Y cell models of excitotoxicity, baicalin showed similar results and significantly promoted PI3K and Akt phosphorylation, rescuing tight junction protein loss and reducing neuronal apoptosis.

### 4.3. Antidepression

Depression is a major mental health-related disease. Different from other diseases, its onset is mainly in mid-to-late adolescence. It prevents people from reaching their full potential; thus, it is prospectively associated with serious issues, including suicide [130]. Currently, depression is conceptualized as a disorder of neural networks, incorporating changes in widely distributed brain areas. Improving synaptic plasticity and modulators of monoamines (e.g., serotonin, noradrenaline, and dopamine) are effective antidepressants [131]. Immune dysfunction is proposed to be relevant in depression. Numerous studies have confirmed the potential role of baicalin in the reversion of depressive-like behaviors. On the one hand, baicalin protects neurons, promoting neuronal survival, proliferation, maturation, and synaptic plasticity [132,133,134,135]. On the other hand, it alleviates neuroinflammation. Associative evidence is strong, with decreased inflammatory cytokine levels, including interleukin IL-β, IL-6, and TNFα, after baicalin administration [136,137]. In addition, several studies have analyzed the potential protective effects of baicalin in improving spatial learning and memory deficits and controlling primary symptoms of attention-deficit hyperactivity disorder (ADHD), which may be influenced by neuroinflammation, but the mechanism needs further investigation [138].

In addition, demyelinating lesions are common pathologic characteristics of various CNS diseases, and baicalin has been reported to promote myelin production and regeneration by activating the peroxisome PPARγ signaling pathway [50]. New progress in the use of baicalin for epilepsy treatment has also been revealed [139].

As discussed above, baicalin may exhibit neuroprotective effects on multiple CNS disorders via mechanisms involving antioxidant stress [140], anti-apoptotic, anti-inflammatory, and anti-excitotoxic effects, ameliorating BBB disruption and promoting neurogenesis, and cell differentiation. As mentioned above, all of this evidence is from preclinical studies, and clinical application needs further concerted effort.

## 5. Regulation of Metabolic Disorders

**Figure 4:**
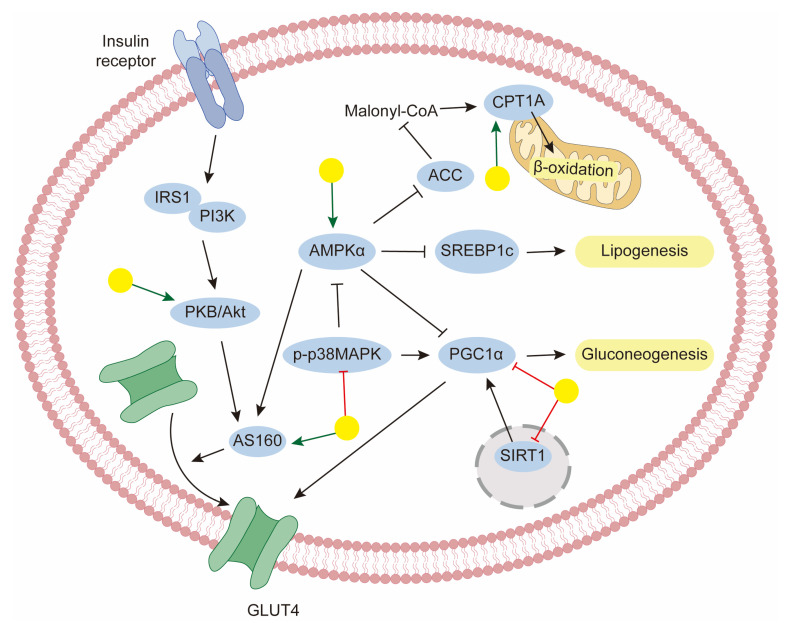
The mechanism of baicalin for the management of metabolic disorders. Yellow balls indicate baicalin.

With changes in lifestyle, metabolic diseases have become a significant burden worldwide. Metabolic diseases include hypertension, type 2 diabetes mellitus (T2DM), hyperlipidemia, obesity, and, more recently, non-alcoholic fatty liver disease (NAFLD). Many of these diseases occur in tandem and share common risk factors [141]. For example, dyslipidemia is a common cause of obesity, hyperlipidemia, and NAFLD. The pathophysiology of T2DM and NAFLD can be largely attributable to insulin resistance. Mounting evidence demonstrates that baicalin is linked to metabolic regulation, which may provide useful hints for the treatment of metabolic diseases [142,143,144,145] (Figure 4). To better understand its pharmacological roles, the metabolic effects are separated in the context of lipid metabolism, glucose homeostasis, and cross-talk pathways involving oxidative stress and inflammation.

### 5.1. Obesity and NAFLD

Obesity, or being overweight, is a condition of excessive fat deposits that presents a risk to health. NAFLD is considered one of the complications of hyperlipidemia and obesity, characterized by the accumulation of toxic lipid species in the liver, which induces hepatocellular stress, injury, and death [146]. Thus, lipid metabolism is closely linked to the etiologies of obesity and NAFLD. Using a system of 3T3-L1 preadipocytes, baicalin was shown to inhibit the differentiation of preadipocytes into adipocytes. Microarray analyses showed that baicalin modulated adipogenesis and cholesterol biosynthesis pathways, and this was confirmed in 3T3-L1 preadipocytes [52]. This result demonstrated the modulation of adipogenesis in adipocytes by baicalin. Its ability in hepatocytes has also been extensively studied. First, we expect to elucidate the pathways leading to lipo-toxicity. Excessive fatty acids serve as substrates for the generation of lipotoxic species. Lipolysis of triglycerides in adipose tissue is the main source of fatty acids in the liver [147]. The second major source of fatty acids is their synthesis from glucose and fructose through de novo lipogenesis, in which acetyl-CoA carboxylase and SREBP-1c play a positive role. In addition, PPARγ ligands enhance the diversion of fatty acids away from the liver [146].

To regulate the processes discussed above, baicalin has shown promise in modulating key transcriptional factors and enzymes through AMP-activated protein kinase (AMPK) activation, which is the central regulator of lipid metabolism. Cellular energy sensor AMPK activation may improve NAFLD by inhibiting lipid and sterol synthesis and stimulating alterations. In this case, baicalin reduced hepatic lipid levels in high-fat diet (HFD)-fed rats, and this protective effect was mainly associated with significant enhancement of hepatic AMPK activation. Furthermore, effects on circulating lipid levels were also confirmed [148]. In a similar study, Li et al. discovered that baicalin ameliorated HFD-caused lipid accumulation in mouse liver models and found that it upregulated the phosphorylation of AMPK at the Thr172 site, and the protective roles of baicalin against NAFLD were exerted through AMPK-mediated modulation of the SREBP1/Nrf2/NF-κB pathways [56]. Fatty acids in the liver were metabolized either via mitochondrial β-oxidation or through esterification to form triglycerides. CPT1 is the rate-limiting enzyme for the fatty acid oxidation process; thus, increasing its level would be a logical strategy for therapy. Wang’s group performed quantitative chemo-proteomic profiling and identify CPT1A as the direct target of baicalin. Decreasing CPT1A activity impaired the anti-steatosis activity of baicalin in vitro. Furthermore, in the DIO animal model, baicalin ameliorated diet-induced obesity and hepatic steatosis. Further disruption of the predicted binding site of baicalin on CPT1A completely abolished the beneficial effect of baicalin [54]. In addition, CPT1 activity seems to be modulated by AMPK activity. Therefore, CPT1A binding and AMPK activation may underlie baicalin’s therapeutic mechanism. There also exists a study about the herb–drug interaction between baicalin and rosuvastatin, which was used for the clinical treatment of dyslipidemia. To a certain extent, baicalin induced hepatic rosuvastatin uptake and decreased rosuvastatin plasma concentrations [149]. Furthermore, baicalin has significant effectiveness in the treatment of NAFLD-related fibrosis and shows potential in hepatoprotective properties [150].

### 5.2. Diabetes

Diabetes is defined as a chronic, metabolic disease characterized by elevated levels of blood glucose, which leads, over time, to serious damage to macrovascular systems, the eyes, kidneys, and nerves. It occurs either when the pancreas does not produce enough insulin or when the body becomes resistant to it. Numerous experiments involving baicalin have shown that it can improve diabetes in preclinical animal models. The anti-diabetic effects of baicalin cover the main insulin-sensitive tissues, such as skeletal muscle, adipose tissue, and the liver.

Controlling hyperglycemia is of great importance for diabetes treatment, which can be achieved through an increase in glucose consumption and the inhibition of gluconeogenesis. In this regard, Wang et al. reported that baicalin decreased plasma glucose levels in a dose-dependent manner in a model of streptozotocin–nicotinamide-induced diabetic rats. In fact, they revealed that the protective properties might be exerted by increasing the hepatic glycogen content and glycolysis [151]. In one study, Wang and coworkers evaluated the glucose consumption level in palmitate induced-insulin resistant HepG-2 cells. They found that baicalin significantly increased glucose consumption and downregulated gluconeogenic genes via the AMPK signaling pathway [152]. Similarly, another study also determined that baicalin suppressed gluconeogenic gene expression in the liver of HFD-fed mice. Moreover, in this study, it was shown that this effect of baicalin was dependent on STAT3 acetylation and activity, regulated by the downregulation of SirT1 [143]. Furthermore, the p38MAPK inhibitor has been shown to strengthen the inhibitory effects of baicalin on glucagon-mediated gluconeogenic gene expression, indicating that baicalin suppresses gluconeogenic activity, at least in part, via the downregulation of p38MAPK [142]. The convergence of multiple pathways suggests that baicalin exerts coordinated regulation of hepatic glucose production.

Another strategy for the treatment of diabetes is the improvement of insulin resistance. It is the decreased sensitivity of target tissues to glucose uptake in response to insulin. In normal conditions, glucose transporter isoform 4 (GLUT4) responds to insulin signals and translocates from the cytoplasm to the cell surface, facilitating the storage of glucose. Several studies have indicated the protective effects of baicalin on the upregulation of GLUT4 levels. Yang et al. found that baicalin promoted glucose disposal in adipocytes dependent on increased AMPK phosphorylation, which subsequently enhanced AS160 phosphorylation, resulting in increased GLUT4 translocation to the plasma membrane [153]. Similar conclusions were also confirmed in myotubes [144,154]. In addition, activated insulin signaling pathways were also reported after baicalin administration in DIO mice [155].

Chronic hyperglycemia leads to complications of diabetes, consisting of microvascular diseases, such as retinopathy, neuropathy, and nephropathy, and macrovascular diseases, including coronary heart disease and cerebrovascular disorders. Excess mitochondrial superoxide production explains the pathobiology of diabetic complications, with inflammation also being involved [156]. Baicalin is an efficient antioxidant through the increased expression of antioxidant enzyme activities in type 2 diabetic rats [157]. In diabetes-associated cognitive impairment, the antioxidant and anti-inflammatory defense of baicalin was mediated through the KEAP1-Nrf2 axis [158]. Its protective effects were also confirmed in hyperglycemia-induced malformation of the cardiovascular system [91] and diabetes-associated kidney disease [58,59]. These studies emphasize the potential of baicalin not only as a hypoglycemic agent but also as a systemic protector against diabetes-related end-organ damage. In addition, one study reported that baicalin suppressed the progression of type 2 diabetes-induced liver tumors through the reversal of high glucose concentration-induced JAK2/STAT1/caspase-3 inhibition [159].

Glucose metabolism and lipid metabolism have a clear and complex biological link. For example, diabetes can lead to disorder in bone–fat balance, and the progression of diabetic nephropathy is usually due to the obstruction of fatty acid oxidation in the renal tubules [59,160]. Baicalin often targets the skeletal muscle, the adipose tissue, and the liver to exert its beneficial effects on glucose and lipid metabolism [145]. In animal models, it has shown great effects in reducing body weight, decreasing hyperglycemia, and mediating dyslipidemia. Taken together, baicalin holds strong translational potential, yet further validation in clinical applications is necessary.

## 6. Discussion

**Figure 5:**
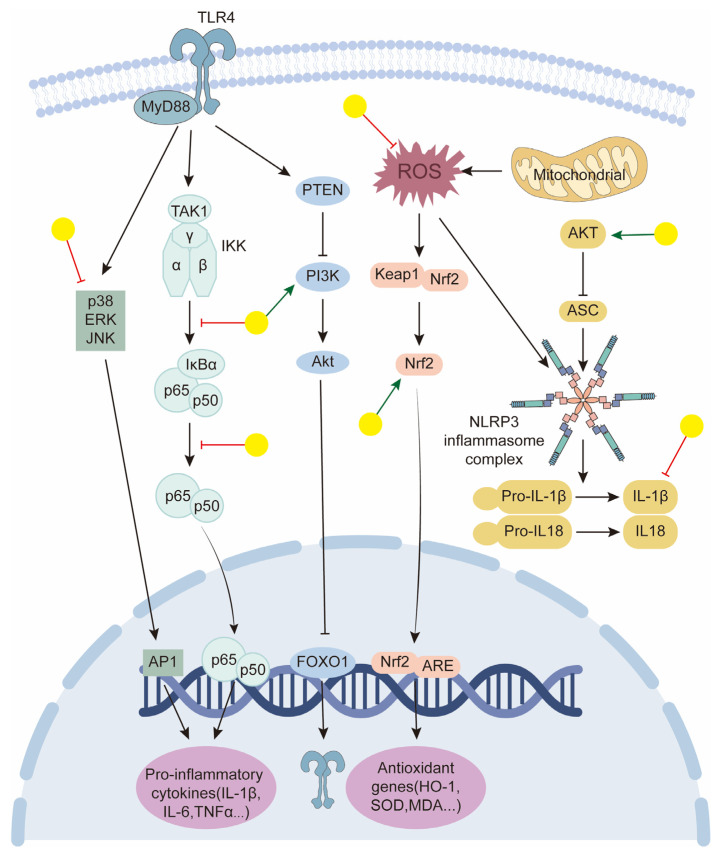
Schematic representation of baicalin targeting in key signaling pathways to promote anti-inflammatory and antioxidant effects. Yellow balls indicate baicalin.

In this review, we summarize the current knowledge regarding the pharmacological effects and associated mechanisms of baicalin in tumors, cardiovascular diseases, and neurological and metabolic disorders. From these studies, we conclude that many of the biological effects of baicalin are attributed to its potent anti-inflammatory and antioxidant capacities [161,162,163] (Figure 5). ROS are closely linked to a variety of oxidative stress-related diseases such as diabetes, Alzheimer’s disease, and Parkinson’s disease. Ferroptosis, a recently discovered type of programmed cell death characterized by the overproduction of ROS [164], is also relevant here. Baicalin can play a protective role in various tissues by scavenging ROS and inhibiting ferroptosis [122,164]. The ortho-dihydroxyl groups in ring A of baicalin contribute significantly to its radical scavenging ability [165,166,167]. As for its anti-inflammatory properties, baicalin modulates various inflammatory signaling pathways, including STING, the NLRP3 inflammasome, TLRs, and NF-κB. Extensive studies have been conducted using different inflammatory disease models, demonstrating that baicalin acts on diverse immune cell types, especially macrophages, T cells, and mast cells. Most experiments have examined the effects on macrophages. Robust evidence supports the anti-inflammatory effect of baicalin in LPS-induced inflammation in macrophages, wherein it decreases the expression of pro-inflammatory proteins and genes [168,169,170]. Similarly, in various animal models of inflammatory diseases, baicalin shows protective effects by modulating the Th17/Treg paradigm, reducing pro-inflammatory cytokine levels, and increasing Treg cell and related cytokine levels [69,171,172]. Mast cells, which play a critical role in allergic reactions, are also influenced by baicalin. For instance, in ovalbumin-induced allergic rhinitis guinea pigs, oral administration of baicalin improved histological changes in the nasal mucosa and decreased serum levels of histamine and other inflammatory markers [64]. The broad anti-inflammatory and antioxidant activities noted in pharmacological studies also indicate its non-selectivity. Conflicting results are observed across different disease models, particularly between tumor models and other disease models. For example, in studies using HCC tumor supernatant-derived TAMs or BMDM-derived macrophages polarized to M1-like or M2-like phenotypes, baicalin treatment skewed M2-like macrophages toward an M1-like phenotype without significantly affecting M1-like macrophages [33]. These findings, along with others showing baicalin-induced M1-type polarization [87], contradict its known anti-inflammatory effects. Regarding ROS, baicalin has been shown to increase ROS levels to inhibit tumors [42] but decrease peroxide-induced oxidative stress to protect tissues [70,140]. This bidirectional regulatory profile suggests that baicalin may act through a complex network of molecular targets. Therefore, we need to further elucidate its differential manifestations across various cells, tissues, and disease states.

To date, most studies have focused on phenotypic observations and explored mechanisms to some extent but lack a systematic exploration of the structure–activity relationship of baicalin. Notably, Wang et al. [54] employed activity-based protein profiling (ABPP), successfully identifying carnitine palmitoyl-transferase 1 (CPT1) as the specific molecular target of baicalin. Based on this finding, they conducted further target-oriented structural modifications, representing a valuable attempt to elucidate its precise molecular mechanisms. From a medicinal chemistry perspective, structure optimization strategies based on molecular target characteristics hold great promise. Molecular design, aimed at enhancing target selectivity, improving pharmacokinetic properties, and reducing toxicity, is a feasible and valuable approach for advancing baicalin as a candidate for innovative drug development.

Moreover, while numerous studies have reported promising biological functions, the majority of them are based on in vitro or murine models. Challenges such as a lack of precise molecular target identification and an incomplete understanding of long-term effects hinder the clinical development of baicalin. This highlights the necessity of critically assessing the current preclinical evidence and the underlying mechanisms. Also, well-designed clinical trials are needed to validate baicalin’s therapeutic efficacy, particularly with regard to key aspects such as oral bioavailability, human pharmacokinetics, and long-term safety. It should be noted that a higher dose of drug exposure is required in vivo to achieve an equivalent effect in vitro due to reasons such as oral bioavailability and drug metabolism. In animal models, various strategies have been proposed to enhance its drug transport and administration methods. Interestingly, a combination of acupuncture with the oral administration of *Scutellaria baicalensis* Georgi extracts significantly improved baicalin absorption in normal rats [173]. Additionally, novel drug delivery systems have attracted attention in the pharmaceutical field. For example, Labrasol, a penetration enhancer, has been shown to increase the corneal permeability and bioavailability of baicalin following topical administration in rabbits [174]. Low-molecular-weight chitosans have also been found to enhance baicalin’s transdermal delivery [175]. Regarding pharmacokinetics, the discrepancy between in vitro and in vivo active forms is often overlooked, and the metabolized components may induce toxicity. Therefore, it is necessary to clearly explain the true components of the drug after metabolism in the body. The toxicity of TCM has always been a concern, with liver injury reported as a proven adverse effect of flavocoxid (which includes baicalin and catechins) [176]. However, another study found no toxicological changes or mortality after oral administration of fermented *Scutellariae radix* extract at a dose of up to 2000 mg/kg in rats or dogs [177]. Experimental data also suggest that baicalin exerts hepatoprotective activity against various hepatotoxic insults, including drug-induced hepatotoxicity [178], liver injury [179,180], hepatic fibrosis [181,182], non-alcoholic steatohepatitis [55], and cirrhosis [183].

Therefore, future studies on baicalin should focus on the following aspects:

- Identifying specific molecular targets in different diseases;
- Exploring the dose–response relationship between baicalin and the tissue-specific response mechanism under multi-organ and multi-pathological conditions to delineate its tissue selectivity;
- Standardizing baicalin preparations, clarifying its pharmacokinetic characteristics, and exploring its application in combination therapies to bridge the gap between bench and bedside;
- Optimizing chemical structure or delivery systems to enhance its oral bioavailability, stability, and overall pharmacological efficacy.

## 7. Conclusions

Baicalin’s versatile beneficial functions in human diseases are increasingly recog-nized, further clinical studies are needed to identify the mechanisms involved in specific human pathological conditions. Moreover, while validating its efficacy through scientific and modern methods, we aspire to unearth its more potent pharmacological actions and, when necessary, employ advanced techniques to structurally modify it, thereby enhancing its value and therapeutic potential.

## Figures and Tables

**Table 1:** Multi-herb formulations containing baicalin.

Name	Active Components or Major Compounds	Application	Reference
Hange-Shashin-To	Baicalin, glycyrrhizin, isoliquiritin, berberine, coptisine, palmitine, and saponins	Diarrhoeal	[2]
Soshiho-tang	Homogentisic acid, baicalin, glycyrrhizin, saikosaponin A, 6-gingerol, and ginsenoside Rg3	Chronic liver disease	[3]
Angong Niuhuang sticker	Curcuma, berberine hydrochloride, baicalin, geniposide, borneol, and musk	Cerebral ischemia	[4]
Huang-Lian-Jie-Du-Tang	Berberine, palmatine, baicalin, baicalein, and gardenoside	Cerebrovascular disease	[5]
Huaijiao pill	Ophoricoside, baicalin, naringin, genistein, rutin, quercetin, and 5-O-methylvisammioside	Hematochezia, edema, and carbuncle	[6]
Gegen Qinlian decoction	Puerarin, liquiritin, berberine, and baicalin	Diarrhea and inflammation symptoms	[7]
Shuanghuanglian preparation	Chlorogenic acid, phillyrin, baicalin, and baicalein	Respiratory tract infection	[8]
Chaiqin chengqi decoction	Emodin, baicalin, rhein, and chrysin	Acute pancreatitis	[9]
Qingda granules	Baicalin	Spontaneous hypertension	[10]
Jinzhen granules	Gallic acid, baicalin, glycyrrhizic acid, hyodeoxycholic acid, and cholic acid	Viral-induced diseases	[11]
Liang-Ge-San	Geniposide, liquiritin, forsythenside A, forsythin, baicalin, baicalein, rhein, and emodin	Virus-induced diseases	[12]
Xiaoer Chaige Tuire oral liquid	Puerarin, daidzein, benzoic acid, baicalin, baicalein, wogonoside, wogonin, oroxylin A, 3′-methoxypuerarin, paeoniflorin, scopoletin, and liquiritigenin	Anti-inflammation and antivirus	[13]
Lanqin oral solution	Geniposide, berberine, palmatine, and baicalin	Pharyngitis	[14]
Qingkailing	Hyodeoxycholic acid, geniposide, baicalin, and cholic acid	Ischemic stroke	[15]
Bu-Shen-Ning-Xin decoction	Berberine, paeoniflorin, morroniside, gallic acid, loganin, and baicalin	Premature ovarian insufficiency	[16]
Wenqingyin	Baicalin, coptisine, and paeoniflorin	Sepsis-induced acute lung injury	[17]
Qing-Yi recipe	Baicalin, wogonoside, geniposide, rhein, costunolide, and paeoniflorin	Acute diseases of the abdomen	[18]
Sanfeng Tongqiao dripping pills	L-Menthone, pulegone, hesperetin, baicalin, wogonin, pulegone, and luteolin	Allergic rhinitis	[19]

**Table 2:** The effects and mechanisms of baicalin.

Disease		Biological Effects	Mechanisms of Action	Reference
Cancer				
	Lymphoma	Induces apoptosis	↓ PI3K/Akt pathway	[32]
	Hepatocellular carcinoma	Repolarization of TAM toward the M1 phenotype	↑ RelB/p52 pathway	[33]
	Colon cancer	Induces senescence	↑ DEPP and Ras/Raf/MEK/ ERK signaling	[34]
	Leukemia	Enhances apoptosis and reduces viability	↓ Akt pathway	[35]
	Bladder cancer	Induces ferroptosis	↓ FTH1	[36]
	Non-small cell lung cancer	Promotes macrophage polarization to the M1 phenotype	↑ JAK2-STAT1 pathway in macrophages	[37]
	Breast cancer	Triggers apoptosis and reduces inflammation and angiogenesis	↓ NF-κB, Bcl-2, VEGF ↑ p53, Bax, and caspase-3	[38]
	Colorectal cancer	Induces apoptosis	↓ CDK/RB	[39]
	Hepatocellular carcinoma	Inhibits proliferation, migration, and invasion and induces cell cycle arrest and apoptosis	↓ ROCK1 signaling	[40]
	Osteosarcomas	Suppresses cell proliferation and induces apoptosis and ferroptosis	↓ Nrf2/ xCT/GPX4 regulatory axis	[41]
	Gastric cancer	Promotes ferroptosis	↑ ROS	[42]
Cardiovascular diseases				
	Atherosclerosis (vascular inflammatory disorders)	Promotes the efflux of cholesterol from macrophages and delays the formation of foam cells	↑ PPARγ-ABCA1/ABCG1 pathway	[43]
	Angiogenesis	-	↑ ERRα pathway.	[44]
	Cardiac hypertrophy and heart failure	-	↑ SIRT3/LKB1/AMPK signaling pathway	[45]
	PAH	-	↑ A (2A) R activity ↓ PI3K/AKT signaling	[46]
	Hypertension	Reduces constriction and enhances vasodilation of abdominal aortic rings	↓ MLCK/p-MLC pathway	[47]
Neurological diseases				
	Parkinson’s disease	Protects dopaminergic neurons	↓ Iron accumulation	[48]
	Alzheimer’s disease	-	↓ COX1-2/5-LOX	[49]
	Demyelinating diseases	Promotes myelin production and regeneration	↑ PPARγ signaling pathway	[50]
	Spinal cord injury	-	↑ PI3K/Akt	[51]
Metabolic diseases				
	Obesity	Modulates the expression of genes in the adipogenesis pathway	↑ Antiadipogenic regulators, including KLF2, C/EBPγ, and CHOP ↓ The proadipogenic regulator KLF15	[52]
	Hepatic steatosis	Decreases serum cholesterol, free fatty acid, and insulinconcentrations↓ Systemic inflammation	↑ AMPK	[53]
	Diet-induced obesity and hepatic steatosis	Antisteatosis	↑ CPT1	[54]
	Non-alcoholic steatohepatitis	Decreases lipid accumulation	↓ SREBP-1c and fatty acid synthase ↑ Fatty acid oxidation enzymes, includingPPARα and CPT1a	[55]
	NAFLD	Decreases lipid accumulation	↑ AMPK and Nrf2 ↓ SREBP1 and NF-κB	[56]
	MAFLD	Oxidative stress and inflammation	↑ p62-Keap1-Nrf2 signaling cascade	[57]
	Diabetic nephropathy	Anti-inflammatory effects	↓ IκB and JAK2 phosphorylation	[58]
	Diabetic kidney disease	Ameliorates renal fibrosis	↑ CPT1α	[59]
Inflammatory diseases				
	Acute pancreatitis (emodinand baicalin)	Anti-inflammatory effects	↓ Serum TNF-a and IL-6 ↓ TLR4	[60]
	Ulcerative colitis	-		
	Anti-asthmatic effects	-	↓ Th17 cells	[61]
	Colitis	Reduces inflammatory mediators	↓ Th17 ↑ Treg cells	[62]
	Asthma	Reduces inflammatory cell infiltration	↓ Phosphodiesterase 4 (PDE4)	[63]
	Allergic rhinitis	Improves allergic rhinitis symptoms and	↓ JAK2-STAT5 and NF-κB signaling	[64]
	Chronic ulcerative colitis	Reduces MPO, NO, and inflammatory cytokine levels	↓ IL-33 expression ↓ NF-κB	[65]
	OA	Alleviates inflammatory injury, increases cell viability, and decreases cell apoptosis	↓ miR-126↓NF-κB	[66]
	Chronic gastritis	Reduces IL-8, IL-1β, TNF-α, PGE2, NO, and ET-1 levels	↓ Akt/NF-κB	[67]
	Lupus	Reduces urine protein levels and ameliorates lupus nephritis	↓ mTOR activation ↓ differentiation of Tfh cells ↑ Expansion of Tfr cells	[68]
	Psoriasis	Decreases the level of inflammatory factors and inhibits Th1/Th17 cell differentiation	↑ PPARγ ↓ Wnt signaling pathway and Th17/IL-17 axis	[69]
	Oral mucositis	Reduces inflammatory storm	↓ oxidative stress and NLRP3	[70]

Note: ↑ activate/upregulate; ↓ inhibit/downregulate. PI3K, phosphatidylinositol-4,5-bisphosphate 3-kinase; TAM, tumor-associated macrophage; RelB, avian reticuloendotheliosis viral (v-rel) oncogene-related B; DEPP, decidual protein induced by progesterone; Raf, rapidly accelerated fibrosarcoma; MEK, mitogen-activated protein kinase Kinase; ERK, extracellular signal-regulated protein kinases; FTH1, ferritin heavy chain 1; JAK, Janus kinase; STAT, signal transducers and activators of transcription; NF-κB, nuclear factor kappa B; Bcl-2, B-cell lymphoma 2; VEGF, vascular endothelial growth factor; Bax, Bcl-2-associated X protein; caspase-3, cysteine-dependent aspartate-specific protease-3; CDK, cyclin-dependent kinase; RB, retinoblastoma protein; ROCK1, Rho-associated coiled-coil containing protein kinase 1; Nrf2, nuclear factor erythroid 2-related factor 2; xCT, Xc-system/SLC7A11; GPX4, glutathione peroxidase 4; ROS, reactive oxygen species; PARP, poly ADP-ribose polymerase; ABCA1, ATP-binding cassette transporter A1; ABCG1, ATP-binding cassette subfamily G member 1; ERRα, estrogen receptor related-α; SIRT, sirtuins; LKB1, liver kinase B1; AMPK, adenosine 5′-monophosphate-activated protein kinase; A(2A)R, α2A-adrenoceptor; PAH, Pulmonary arterial hypertension; MLCK, myosin light chain kinase; p-MLC, phosphorylated myosin light chain; COX, cyclooxygenase; LOX, lipoxygenase; KLF, Krüppel-like factor; C/EBP, CCAAT enhancer binding protein; CHOP, CCAAT enhancer-binding protein homologous protein; CPT1, carnitine palmitoyl-transferase1; SREBP-1c, sterol regulatory element binding protein 1c; PPAR, peroxisome proliferator-activated receptor; Keap1, Kelch-like ECH-associated protein 1; IκB, nuclear factor of kappa light polypeptide gene enhancer in B-cells inhibitor; TNF, tumor necrosis factor; IL-6, interleukin-6; TLR, Toll-like receptors; Th17, T helper cell 17; Treg, regulatory T cells; PDE4, phosphodiesterase 4; MPO, myeloperoxidase; NO, nitric oxide; OA, osteoarthritis; miR-126, microRNA-126; PGE2, prostaglandin E2; ET-1, endothelin-1; mTOR, mammalian target of rapamycin; Tfh, follicular helper T cell; Tfr, T follicular regulatory Cells; Wnt, wingless/integrated; NLRP3, nod-like receptor, pyrin domain containing 3.

## Data Availability

Not applicable.

## Abbreviations

The following abbreviations are used in this manuscript:

TCM	traditional Chinese medicine
HCC	hepatocellular carcinoma
TME	tumor microenvironment
TAM	tumor-associated macrophage
ROS	reactive oxygen species
CVDs	cardiovascular diseases
H/R	hypoxia/reoxygenation
IR	ischemia–reperfusion
Ang II	angiotensin II
AAC	abdominal aortic constriction
VSMCs	vascular smooth muscle cells
PDGF	platelet-derived growth factor
VEGF	vascular endothelial growth factor
VDCCs	voltage-dependent Ca^2+^ channels
BK	large-conductance Ca^2+^-activated channels
BBB	Blood–brain barrier
NMDA	*N*-methyl-d-aspartate
NOS	nitric oxide synthase
GS	glutamine synthetase
pMCAO	permanent middle cerebral artery occlusion
TNFα	tumor necrosis factor α
AD	Alzheimer’s disease
PD	Parkinson’s disease
CNS	central nervous system.
BSCB	blood–spinal cord barrier
PPAR	peroxisome proliferator-activated receptor
T2DM	type 2 diabetes mellitus
NAFLD	non-alcoholic fatty liver disease
SREBP-1c	sterol-CoA response element binding protein-1c
AMPK	AMP-activated protein kinase
HFD	high-fat diet
CPT1	carnitine palmitoyl-transferase1
GLUT4	glucose transporter isoform 4
DARTS	drug affinity responsive target stability
